# A systematic review and meta-analysis of randomised controlled trials of surgical treatments for ingrown toenails part II: healing time, post-operative complications, pain, and participant satisfaction

**DOI:** 10.1186/s13047-023-00655-7

**Published:** 2023-09-06

**Authors:** Victoria Exley, Katherine Jones, Grace O’Carroll, Judith Watson, Michael Backhouse

**Affiliations:** 1 York Trials Unit, Department of Health Sciences, University of York, York, UK; 2 Warwick Clinical Trials Unit, Warwick Medical School, University of Warwick, Coventry, CV4 7AL UK

**Keywords:** Ingrown nails, Nail, Malformed, Onychocryptosis, Nail surgery, Nail avulsion, Review, Systematic

## Abstract

**Background:**

When performing nail surgery, clinicians must choose from a multitude of procedures and variations within each procedure. Much has been published to guide this decision making, but there are a lack of up to date robust systematic reviews to assess the totality of this evidence.

**Methods:**

Five databases (MEDLINE, Embase, CINAHL, Web of Science and CENTRAL) and two registers (Clinicaltrials.gov and ISRCTN) were searched to January 2022 for randomised trials evaluating the effects of a surgical intervention(s) for ingrown toenails. Two independent reviewers screened records, extracted data, assessed risk of bias and certainty of evidence. Data on co-primary outcomes of symptom relief and symptomatic regrowth were presented in our first paper. This paper presents data for the secondary outcomes and further discussion.

**Results:**

Of 3,928 records identified, 36 randomised trials were included in the systematic review. Healing time appears to be reduced with shorter application of phenol. A reduced healing time was also apparent was with the addition of curettage, although this may also increase the risk of post-operative bleeding and pain. Post operative bleeding was also reportedly lower in people who received local anaesthetic with epinephrine but no tourniquet. Use of phenol with nail bed excision may decrease the risk of infection. Lower pain scores were reported when using partial matrixectomy and surgical interventions with phenol. Shorter duration of pain was reported with phenolisation and wedge resection. Participant satisfaction was high overall.

**Conclusion:**

This second paper reports secondary outcomes from a robust systematic review of randomised trials on surgical treatment of ingrown toenails. Despite the large volume of clinical trials conducted on the topic, few clinical conclusions can be drawn due to the poor quality of these studies. Further high-quality clinical trials are needed to answer fundamental questions in the surgical treatment of ingrown toenails.

**Supplementary Information:**

The online version contains supplementary material available at 10.1186/s13047-023-00655-7.

## Introduction

Ingrown toenails (onychocryptoses) are one of the most common nail pathologies. It has been suggested that they have a prevalence of between 2.5% and 5% with a bimodal distribution of age with peaks at 15 and 50 years [1, 2]. Patients typically present with pain as their main symptom and this can often cause difficulty with footwear and walking [3]. As the nail plate pierces the periungual tissue, it leads to local inflammation and frequently secondary bacterial infection with associated serosanguinous or purulent discharge [3]. Over time, this can become chronic as the nail plate continues to grow causing hypergranulation tissue to proliferate and protrude from the nail sulcus [4].

Mild early cases can often be treated with conservative interventions, but many cases require some form of nail surgery. Indeed, nail surgery is so frequently performed that it has been identified as the tenth most common procedure performed by podiatrists [5]. Although there are multiple procedures and options on how to perform such surgery, it typically aims to remove the problem part of the nail and destroy the underlying matrix to avoid recurrence [6–8]. As with many interventions in healthcare, nail surgery can be considered a complex intervention as it contains multiple interacting components that often need tailoring to the needs of individual patients [9–11]. When deciding on each of these components for a given patient, clinicians must make multiple decisions based on the available evidence.

A large number of papers have been published on the treatment of ingrown toenails including many narrative or scoping reviews, but with the most recent Cochrane review now over a decade old, there is a lack of current high quality systematic reviews and meta-analyses [7, 12–14]. The authors therefore aimed to systematically search and synthesise the literature relating to the effectiveness/efficacy of surgical methods for treating ingrown toenails. Given the volume of studies and data on this topic, the review has been split into two parts with the linked paper reporting in detail the results from the co-primary outcomes of recurrence and relief of symptoms[15]. This paper presents the secondary outcomes: healing time, post-operative complications (infection and haemorrhage), pain of operation/ post-operative pain and participant satisfaction.

## Methods

The conduct and reporting of this review were guided by the Cochrane Handbook for Systematic Reviews of Interventions [16] and the Preferred Reporting Items for Systematic Reviews and Meta-Analyses (PRISMA) [17]. It was prospectively registered [PROSPERO: CRD42021251938].

### Eligibility criteria

Randomised controlled trials (RCT) were included if they evaluated the effects of a surgical intervention(s) for ingrown toenails with a follow-up period of at least one month. Our inclusion criteria were broad, and we did not restrict eligibility based on the trial setting, age, or gender of participants. Studies were restricted to English, pertaining to human participants, and must have reported one of the pre-defined outcomes for inclusion. Our co-primary outcomes were relief of symptoms, and symptomatic regrowth (nail spicules/nail spikes), which are reported elsewhere [15]. Secondary outcomes: healing time, post-operative complications (e.g., infection and haemorrhage), pain of operation, post-operative pain (duration and intensity) and participant satisfaction are reported herein.

### Search strategy

We searched MEDLINE (Ovid), EMBASE (Ovid), CINAHL, Web of Science and Cochrane Central Register of Controlled Trials (CENTRAL) from inception to January 2022 using a multifaceted search strategy (Supplementary File 1). We also searched trial registers (International Clinical Trials Registry and Clinicaltrials.gov) and forward and backward citations of included studies.

Results were exported into Rayyan for de-duplication. Title, abstract, and full text screening were conducted independently by two reviewers and any discrepancies were assessed by a third reviewer and resolved by consensus.

### Data extraction

Two reviewers independently extracted data using a modified Cochrane data extraction form. Disagreements were resolved through consensus with a third author. Data extracted included: (a) general information such as author(s), title, journal and study funding; (b) trial characteristics such as study aim and objectives, study design, unit of allocation and ethical approvals; (c) participant characteristics such as setting, inclusion/exclusion criteria, sample size (number of participants and nail folds), age, gender, baseline imbalances, severity of ingrown toenails; (d) intervention and comparison group(s); (e) outcome measures including as time points, unit of measurement, outcome definition, data at baseline/follow-up and statistical methods. Where data were missing or unclear, clarification was sought via email to the corresponding author(s). At least one follow-up email was sent if a response was not forthcoming.

### Risk of bias

The Cochrane Risk of Bias tool (RoB 2.0) was used to evaluate risk of bias [18].

### Data synthesis and analysis

Despite, intending to conduct meta-analyses on these secondary outcomes, unfortunately this was not possible due to a combination of studies not conducting intention to treat analysis, poor reporting, heterogeneity in the intervention and timepoints at which the outcomes were captured, and unavailability of data. Thus, these secondary outcomes could only be reported narratively, and conclusions are therefore limited.

## Results

The PRISMA flow chart (Supplementary File 2), study characteristics (Supplementary Table 1) and interventions are detailed in paper 1[15].

### Healing time

Time to healing was assessed in 14 studies [19–32] (Table 1). The definition of healing varied between studies (Table 2) and was only provided in seven. Follow-up ranged from 1 to 24 months, though the exact timepoint each outcome was captured was not always clear. Findings suggest shorter application time for chemical matrixectomy with phenol resulted in a faster healing time [26, 28], as did the addition of curettage [32].

**Table 1:** Outcome measure: Healing Time (*n* = 14)

**Author (Year)**	**Interventions**	**Timepoint**	**Healing Time** (mean ± SD)*	**Significance** (*p* value)
*Conservative treatment (e.g., braces and gutter treatment) v’s Chemical matrixectomy (n* = *1 study)*				
AlGhamdi (2014) [19]	A: Lateral nail avulsion with phenol (*n* = 30)	1, 3 and 6 months	Not reported	The healing period ranged from 1–2 weeks. No statistical analysis reported
	B: Nail tube splinting (*n* = 23)		Not reported	
*Chemical matrixectomy v’s Surgical matrixectomy (n* = *4 studies)*				
Varma (1983) [29]	A: Surgical wedge excision (*n* = 35)	1 week, 1 month, 3 months and 6 months	Average of 2 weeks to heal	No statistical analysis reported
	B: Phenol wedge cauterisation (*n* = 28)		Average of 2 weeks to heal	
Van der Ham (1990) [28]	A: Wedge excision (*n* = 124)	Seven days and then at weekly intervals until the wound had healed	2.5 weeks no SD reported	No statistical analysis reported
	B: Segmental phenol cauterisation (*n* = 125)		2.2 weeks no SD reported	
Akkus (2018) [18]	A: Chemical matrixectomy with NaOH (*n* = 30)	Healing time assessed at day 3, Week 1, Month 1, 6, and 12	17.3 ± 14.2 days	*p* = 0.040
	B: Wedge resection (*n* = 30		28.8 ± 17 days	
Muriel-Sánchez (2020) [24]	A: Chemical matrixectomy with phenol (*n* = 10)	The period of time between the surgical action and the solving of the draining and/or inflammatory changes	21.3 ± 3.1 days [95% CI 20.20 to 22.39]	*p* < 0.001
	B: “Aesthetic reconstruction” (describes partial nail ablation with wedge excision of matrix) (*n* = 24)		8.2 ± 1.4 days [95% CI 7.92 to 8.55]	
*Chemical v’s Other chemical (n* = *1 study)*				
Gem (1990) Study 1[21]	A: Chemical ablation with 3-min application of 80% phenol (*n* = 109)	Until healing occurred	The average time to complete healing was 40 days, again with no statistical difference between the groups	No statistical analysis information provided
	B: Chemical ablation with 2-min application of 10% sodium hydroxide (*n* = 110)		The average time to complete healing was 40 days, again with no statistical difference between the groups	
*Chemical timings (n* = *3 studies)*				
Gem (1990) Study 2[21]	A: Chemical ablation with 2-min application of 10% sodium hydroxide (*n* = 110)	Until healing occurred	The average time to complete healing was 40 days, again with no statistical difference between the groups	No statistical analysis information provided
	B: Chemical ablation with 1-min application of 10% sodium hydroxide (*n* = 93)		The average time to complete healing was 40 days, again with no statistical difference between the groups	
Tatlican (2009) [28]	A: Partial nail avulsion with 1 min phenol cauterisation (*n* = 37)	Patients were examined on alternate days until the complete healing was achieved	13.5 ± 3.9 days	A vs B = *p* = < 0.001
	B: Partial nail avulsion with 2-min phenol cauterisation (*n* = 36)		17.5 ± 2.8 days	A vs C = *p* = < 0.001
	C: Partial nail avulsion with 3-min phenol cauterisation (*n* = 37)		17.1 ± 2.6 days	B vs C = *p* = 0.853
Muriel-Sánchez (2021) [27]	A: Partial nail avulsion with 30 s application of phenol (*n* = 27 halluces [54 nail folds])	Until healing was achieved	14.9 ± 2.8 days	*p* < 0.001
	B: Partial nail avulsion with 60 s application of phenol (*n* = 27 halluces [54 nail folds])		22 ± 3.2 days	
*Chemical matrixectomy v’s Surgical* + *chemical matrixectomy (n* = *1 study)*				
Alvarez-Jimenez (2011) [31]	A: Phenol and curettage (*n* = 73 nail folds)	1 month (digital photographs)	7.5 ± 1.8 days	*p* = 0.001
	B: Phenol (*n* = 79 nail folds)		12.4 ± 3 days	
*Chemical matrixectomy v’s ‘Other’ (e.g., laser and electrocautery) (n* = *1 study)*				
Misiak (2014) [23]	A: Partial nail extraction + phenolisation (*n* = 30)	10 days	*n* = 10/30 (33.3%)	OR 4.5 [95% CI 1.09 to 18.50 *p* = 0.020)
	B: Partial nail extraction + electrocautery (*n* = 30)		*n* = 3/30 (10%)	
*Surgical matrixectomy v’s ‘Other’ (e.g., laser and electrocautery) (n* = *2 studies)*				
Kavoussi (2020) [22]	A: Partial Nail Matrixectomy using CO_2_ laser (*n* = 62)	Timepoint unclear. Participants were followed over 24 months	13 ± 2.5 days	*p* = 0.620
	B: Lateral Nail Fold Excision (LNFE) (*n* = 65)		12.2 ± 2.2 days	
Awad (2020) [20]	A: Partial nail matrixectomy with electrocautery (*n* = 100)	3^rd^ and 7^th^ day, 1 and 6 months	> 12 days: *n* = 51 (25.5%) 12 days: n = 49 (24.5%)	*p* = 0.02^*a*^
	B: Partial nail matrixectomy (*n* = 100)		> 12 days: *n* = 53 (26.5%) 12 days: n = 47 (23.5%)	
*Antibiotics (n* = *1 study)*				
Reyzelman (2000) [26]	A: 1 week course of oral antibiotics and simultaneous phenol matrixectomy (*n* = 53)	Until healing occurred	1.9 ± 0.7 weeks	Group A healed significantly sooner than group B (*P* < 0.04). No further information is provided
	B: 1 week course of oral antibiotics and phenol matrixectomy 1 week later (*n* = 51)		2.3 ± 0.8 weeks	
	C: Phenol matrixectomy without antibiotic therapy (*n* = 50)		2.0 ± 0.8 weeks	

*SD* Standard Deviation, *CI* Confidence Interval, *OR* Odds Ratio, *NaOH* Sodium Hydroxide

^*^Unless otherwise specified

^*a*^Unclear on the timepoints included in the analysis

**Table 2:** Healing definitions

Akkus (2018) [18]	No definition provided
AlGhamdi (2014) [19]	No definition provided
Altinyazar (2010) [30]	No definition provided
Alvarez-Jimenez (2011) [31]	The clinical criteria of early healing time were considered to be absence of drainage (no exudate evident), granulation tissue covered by a scab (no evidence of hyper granulation tissue), and no signs of infection (i.e. pain and clinical evidence of discharge in association with redness extending proximally). The patient was then allowed to bathe. All criteria had to be met before the wound was considered cicatrized (healed)
Awad (2020) [20]	Healing was inspected for the complete re-epithelialization of nail bad and regression of edema
Gem (1990) a [21]	No definition provided
Gem (1990) b [21]	No definition provided
Kavoussi (2020) [22]	No definition provided
Misiak (2014) [23]	Healing was defined as the complete reepithelialization of nail bed, regression of oedema and cessation of discharge
Muriel-Sánchez (2020) [24]	The healing time was measured paying attention to the previously described criteria, considering it to be the period of time between the surgical action and the solving of the draining and/or inflammatory changes. The criteria are absence of exudate in the gauze; the forming of a scab which covers the granulation tissue; the wound must be kept uncovered; a lack of signs of infection or inflammation in the zone operated; there are no signs of erythematosus tissue or of hyper granulation
Muriel-Sánchez (2021) [25]	The healing time was measured as previously described criteria, considering the period of time between ending surgical procedure and resolution of the postoperative period. These criteria were absence of exudate at gauze; formation of scab covering the wound; the wound must be kept uncovered; no signs of infection or inflammation at nail folds; no signs of erythema or hypergranulation tissue
Reyzelman (2000) [26]	Healing time was defined as the interval between the day the phenol matrixectomy was performed and the resolution of drainage and inflammatory changes surrounding the nail border. In every case, healing was identified by the principal investigator of the trial
Tatlican (2009) [27]	Complete healing was defined as the complete reepithelialization of the nail bed and the cessation of drainage
Van der Ham (1990) [28]	No definition provided
Varma (1983) [29]	No definition provided

### Post-operative complications (Infection & Haemorrhage)

Twenty studies [20, 21, 23, 25–27, 32–45] (Table 3) assessed the post-operative complications of infection and/or haemorrhage. Follow-up times varied across the studies, ranging from just 48 h for haemorrhage [32], to 3 to 5 days for infection [45] and some up to 6 months [42] and beyond. It was also often unclear which outcome had been collected at which timepoint, and measurement techniques were often unclear or poorly reported. Of the 20 studies, only 2 studies [33, 36] mention the use of bacterial cultures to identify infective organisms and 2 studies [25, 44] reported measuring post-operative bleeding using a scale of mild, moderate or abundant on assessment of the dressing.

**Table 3:** Outcome measure: Post-operative complications (infection and haemorrhage) (*n* = 20)

**Author (Year)**	**Interventions**	**Outcome**	**Timepoint**	**Complication Scores** (mean ± SD)*	**Significance** (*p* value)
*Conservative treatment (e.g., braces and gutter treatment) v’s Chemical matrixectomy (n* = *1 study)*					
AlGhamdi (2014) [19]	A: Lateral nail avulsion with phenol (*n* = 30)	Infection	Timepoint unclear	No infections noted in either group	No statistical analysis reported
	B: Nail tube splinting (*n* = 23)			No infections noted in either group	
*Conservative treatment (e.g., braces and gutter treatment) v’s surgical matrixectomy (n* = *2 studies)*					
Kruijff (2008) [39]	A: Partial nail extraction with partial matrix excision (*n* = 58)	‘Post-operative morbidity’ looking at redness, purulent exudate and post-operative bleeding	1 week	Redness: *n* = 32 (55.2%) Exudate: *n* = 10 (17.2%) Post-Operative Bleeding: n = 5 (8.6%)	Redness: *p* < 0.001 Exudate: *p* = 0.030 Post-Operative Bleeding: *p* = 0.060
	B: Orthonyxia (*n* = 51)			Redness: *n* = 5 (9.8%) Exudate: *n* = 2 (3.9%) Post-Operative Bleeding: n = 0 (0%)	
Peyvandi (2011) [41]	A: Winograd method (*n* = 50)	Infection	1 week, 1 month and 6 months (telephone calls and visits)	1 week: 1 (2%) 1 month: 2 (4%) 6 months: 0	No statistical analysis reported
	B: Sleeve (gutter) method (*n* = 50)			1 week: 1 (2%) 1 month: 3 (6%) 6 months: 0	
*Chemical matrixectomy v’s Surgical matrixectomy (n* = *4 studies)*					
Leahy (1990) [40]	A: Chemical ablation (phenol) (*n* = 32)	Infection and haemorrhage	Patients were examined at 1 week, 3 months, and be- tween 16 and 30 months after surgery by an independent observer	Infection: *n* = 4 Haemorrhage: *n* = 1	No statistical analysis reported
	B: Surgical ablation (*n* = 34)			Infection: *n* = 3 Haemorrhage: *n* = 1	
Bos (2007) [34]	A: Partial avulsion with excision of the matrix, no antibiotics (*n* = 38)	Infection	2 days and 1 week	2 days: Not reported 1 week: 19 of 38	Antibiotics (A vs B) 2 days: *p* = 0.989 1 week: *p* = 0.676 Phenol (C vs D) 2 days: *p* = 0.224 1 week: *p* = 0.501
	B: Partial avulsion with excision of the matrix, with antibiotics (*n* = 22)			2 days: Not reported 1 week: 10 of 21	
	C: Partial avulsion with application of phenol, no antibiotics (*n* = 37)			2 days: Not reported 1 week: 19 of 33	
	D: Partial avulsion with application of phenol, with antibiotics (*n* = 26)			2 days: Not reported 1 week: 13 of 25	
Korkmaz (2013) [38]	A: Partial matrix excision (*n* = 17)	Complications including infection	Timepoint unclear	In both groups, none of the patients had postoperative complications	*p* = 0.688
	B: Segmental phenolisation (*n* = 22)			In both groups, none of the patients had postoperative complications	
Muriel-Sánchez (2020) [24]	A: Chemical matrixectomy with phenol (*n* = 10)	Post-operative bleeding (mild = 1, moderate = 2 and abundant = 3) and infection	The intensity of the bleeding came from the photographic assessment carried out during the first dressing	Bleeding: 1.67 ± 0.58 (95% CI 1.48 to 1.86) Infection: Two incidences	Bleeding:* p* = 0.910 Infection: *p* = 0.820
	B: “Aesthetic reconstruction” (describes partial nail ablation with wedge excision of matrix) (*n* = 24)			Bleeding: 1.65 ± 0.62 (95% CI 1.51 to 1.79) Infection: Two incidences	
*Chemical v’s Other chemical (n* = *2 studies)*					
Andre (2018) [33]	A: Nail avulsion and phenol (*n* = 46 toenails)	Oozing (‘present’ or ‘absent’) and Inflammation (‘present’ or ‘absent’ and on a scale of 0–3)	Day 2, 2 and 4 weeks, 4 months	Oozing Day 2: present in 89.4% Week 2: present in 35.1% Week 4: present in 9.4% Month 4: Not present Inflammation Day 2: 28.3% scoring 0, 43.4% scoring 1, 23.9% scoring 2, 4.3% scoring 3 Week 2: 54.3% scoring 0, 34.3% scoring 1, 5.7% scoring 2, 5.7% scoring 3 Week 4: 83.3% scoring 0, 16.7% scoring 1, 0% scoring 2, 0% scoring 3 Month 4: Not present	Oozing Day 2: *p* = 0.200 Week 2: *p* < 0.010 Week 4: *p* < 0.010 Inflammation Day 2: *p* = 0.340 Week 2: *p* = 0.520 Week 4:* p* = 0.030
	B: Nails avulsion and trichloroacetic acid (*n* = 50 toenials)			Oozing Day 2: present in 97.8% Week 2: present in 77.8% Week 4: present in 39.4% Month 4: Not present Inflammation Day 2: 17% scoring 0 40.4% scoring 1, 40.4% scoring 2, 2.1% scoring 3 Week 2: 38.9% scoring 0, 47.2% scoring 1, 11.1% scoring 2, 2.8% scoring 3 Week 4: 54.5% scoring 0, 36.4% scoring 1, 3% scoring 2, 6.1% scoring 3 Month 4: Not present	
Ahsan (2019) [42]	A: Chemical matrixectomy with phenol (*n* = 50)	Infection	Not clear	Present (*n* = 14) Absent (*n* = 33)	*p* = 0.306
	B: Chemical matrixectomy with trichloroacetic acid (*n* = 50)			Present (*n* = 9) Absent (*n* = 35)	
*Chemical timings (n* = *1 study)*					
Muriel-Sánchez (2021) [25]	A: Partial nail avulsion with 30 s application of phenol (*n* = 27 halluces [54 nail folds])	Post-operative bleeding (mild = 1, moderate = 2 and abundant = 3), inflammation (flexible ruler) and infection	The intensity of the bleeding came from the photographic assessment carried out during the first dressing	Bleeding: 1.7 ± 0.5 [CI 95% 1.50 to 1.90] Inflammation: 0.2 ± 0.5 [CI 95% 0.12 to 0.28] Infection: One incidence	Bleeding: *p* = 0.590 Inflammation: *p* = 0.470 Infection: *p* = 0.480
	B: Partial nail avulsion with 60 s application of phenol (*n* = 27 halluces [54 nail folds])			Bleeding: 1.6 ± 0.5 [CI 95% 1.60 to 1.84] Inflammation: 0.3 ± 0.3 [CI 95% 0.18 to 0.42] Infection: One incidence	
*Chemical matrixectomy v’s Surgical* + *chemical matrixectomy (n* = *1 study)*					
Alvarez-Jimenez (2011) [31]	A: Phenol and curettage (*n* = 73 nail folds)	Post-operative bleeding (light, moderate or abundant) and infection	Bleeding- 48 h Infection- 1 month	Bleeding: Abundant 30 (42.9%) participants, light/moderate 30 (42.9%) Infection: 2 (2.7%)	Bleeding: *p* < 0.001 Infection: *p* = 0.010
	B: Phenol (*n* = 79 nail folds)			Bleeding: Abundant 4 (5.4%) participants, light/moderate 70 (94.6%) Infection: 13 (16.5%)	
*Chemical matrixectomy v’s ‘Other’ (e.g., laser and electrocautery) (n* = *1 study)*					
Hamid (2021) [36]	A: Partial nail avulsion and matrixectomy with phenol (*n* = 50)	Serous and purulent discharge	4 and 6 weeks	Serous discharge: 2 participants Purulent discharge: 2 participants	Serous discharge: *p* = 1.00 Purulent discharge: *p* = 1.00
	B: Partial nail avulsion and matrixectomy with electrocautery (*n* = 50)			Serous discharge: 1 participant Purulent discharge: 2 participants	
*Surgical matrixectomy v’s Surgical* + *chemical matrixectomy (n* = *1 study)*					
Anderson (1990) [32]	A: Nail bed excision (*n* = 17)	Infection	2 weeks post-surgery	Seven occurrences	*p* < 0.010
	B: Combination of nail bed phenolisation and excision (*n* = 14)			Two occurrences	
*Surgical matrixectomy v’s ‘Other’ (e.g., laser and electrocautery) (n* = *2 studies)*					
Kim (2015) [44]	A: Curettage (*n* = 32)	Infection	3–5 days post procedure	Five (15.6%) occurrences	*p* = 0.710
	B: Electrocautery (*n* = 29)			Three (10.3%) occurrences	
Kavoussi (2020) [22]	A: Partial Nail Matrixectomy using CO_2_ laser (*n* = 62)	Infection	Timepoint unclear. Participants were followed over 24 months	Three (4.8%) occurrences	*p* = 0.485
	B: Lateral Nail Fold Excision (LNFE) (*n* = 65)			Two (3.1%) occurrences	
*Surgical v’s Surgical (n* = *1 study)*					
Uygur (2016) [52]	A: Winograd procedure and new suturing technique (*n* = 64)	Antibiotic administration	Timepoint unclear	Five participants required antibiotics	No statistical analysis provided
	B: Winograd procedure and traditional suturing technique (*n* = 64)			Nine participants required antibiotics	
*Chemical matrixectomy v’s Avulsion only (n* = *2 studies)*					
Greig (1991) [35]	A: Total avulsion (*n* = 81 nail edges)	Infection	2 weeks	No occurrences	No statistical analysis provided
	B: Nail edge excision (*n* = 56 nail edges)			One (2%) occurrence	
	C: Nail edge excision and phenolisation (*n* = 67 nail edges)			Seven (12%) occurrences	
Khan (2014) [37]	A: Partial Nail Avulsion + Phenol (*n* = 50)	Infection	3^rd^ and 7^th^ day	4% of participants experienced an occurrence	*p* = 0.029
	B: Partial Nail Avulsion alone (*n* = 50)			12% of participants experienced an occurrence	
*Anaesthetics (with and without epinephrine) (n* = *1 study)*					
Cordoba-Fernandez (2015) [43]	A: Segmental phenolisation matrixectomy with anaesthetic digital block with epinephrine (*n* = 34 toes)	Bleeding (‘light’, ‘moderate’ and ‘abundant’)	Timepoint unclear	17.65% (7/36) of toes presenting abundant bleeding	*p* = 0.001
	B: Segmental phenolisation matrixectomy with anaesthetic digital block without epinephrine (36 toes)			94.4% (34/36) of toes presenting abundant bleeding	
*Antibiotics (n* = *2 studies)*					
Reyzelman (2000) [26]	A: 1 week course of oral antibiotics and simultaneous phenol matrixectomy (*n* = 53)	Infection	Timepoint unclear	Not reported	No significant difference in the prevalence of post procedure infections between groups
	B: 1 week course of oral antibiotics and phenol matrixectomy 1 week later (*n* = 51)			Not reported	
	C: Phenol matrixectomy without antibiotic therapy (*n* = 50)			2 post procedure infection	
Bos (2007) [34]	A: Partial avulsion with excision of the matrix, no antibiotics (*n* = 38)	Infection	2 days and 1 week	2 days: Not reported 1 week: 19 of 38	Antibiotics (A vs B) 2 days: *p* = 0.989 1 week: *p* = 0.676 Phenol (C vs D) 2 days: *p* = 0.224 1 week: *p* = 0.501
	B: Partial avulsion with excision of the matrix, with antibiotics (*n* = 22)			2 days: Not reported 1 week: 10 of 21	
	C: Partial avulsion with application of phenol, no antibiotics (*n* = 37)			2 days: Not reported 1 week: 19 of 33	
	D: Partial avulsion with application of phenol, with antibiotics (*n* = 26)			2 days: Not reported 1 week: 13 of 25	

*SD* Standard Deviation, *CI* Confidence Interval, *OR* Odds Ratio, *NaOH* Sodium Hydroxide

^*^Unless otherwise specified

^a^Unclear on the timepoints included in the analysis

Few studies reported any statistically significant findings. Redness and exudate was found to be reduced when comparing nail bracing to matrix excision [46] and the use of phenol over trichloroacetic acid appeared to reduce oozing at week two and four [34]. The addition of curettage to chemical matrixectomy [32] increased bleeding but showed lower infection rates. Two studies [33, 38] found the addition of phenol led to significantly lower infection rates.

### Pain of operation / Post-operative pain

Post-operative pain was reported in 25 studies [19–23, 25, 26, 28, 29, 31, 32, 34, 37–41, 43, 44, 46–51] (Table 4) and was the second most frequently reported outcome after recurrence. Ten studies [21, 25, 26, 32, 34, 38, 40, 44, 47, 50] measured pain using a Visual Analogue Score (VAS), two studies used a Linear Analogue Score [49, 52], three studies classified pain as mild, moderate or severe [19, 39, 43] and one study assessed analgesic usage [48]. The remaining studies were unclear. Follow-up times varied throughout the studies and ranged from 2 days to 12 months. Few studies reported any significant findings for this outcome. Nail bracing was found to have higher pain levels than matrix excision at 4 and 26 weeks but no difference at 12 weeks [46]. Two studies [29, 52] found pain duration to be shorter with chemical matrixectomy than excision.

**Table 4:** Outcome measure: Pain of operation / Post-operative pain (*n* = 25)

**Author (Year)**	**Interventions**	**Timepoint and pain type (measure)**	**Pain Scores** (mean ± SD)*		**Significance** (*p* value)
*Conservative treatment (e.g., braces and gutter treatment) v’s Chemical matrixectomy (n* = *2 studies)*					
AlGhamdi (2014) [19]	A: Lateral nail avulsion with phenol (*n* = 30)	Time that post-operative pain lasted. Timepoint unclear	29.48 h		*p* = 0.057
	B: Nail tube splinting (*n* = 23)		21.91 h		
Ceren (2013) [50]	A: Partial nail extraction with phenol matrixectomy (*n* = 63 toenails)	Pre- and post-operative pain at 2 days, 1- and 6-months post procedure	Not reported		Postoperative pain scores were lower than preoperative scores in both groups (*p* < .001)^*a*^
	B: Partial nail elevation and flexible tube (57 toenails)		Not reported		
*Conservative treatment (e.g., braces and gutter treatment) v’s surgical matrixectomy (n* = *1 study)*					
Kruijff (2008) [45]	A: Partial nail extraction with partial matrix excision (*n* = 58)	4, 12 and 26 weeks (scale of 1–10) post-operative pain	4 weeks: 5.74* 12 weeks: 7.65* 26 weeks: 5.64*		4 weeks: *p* = 0.010 12 weeks: *p* = 0.060 26 weeks: *p* < 0.010
	B: Orthonyxia (*n* = 51)		4 weeks: 8.11* 12 weeks: 9.74* 26 weeks: 9.62*		
*Chemical matrixectomy v’s Surgical matrixectomy (n* = *9 studies)*					
Morkane (1984) [48]	A: Segmental or angular phenolisation (*n* = 54)	1 week (10 cm linear analogue scale)	20.72 mm (± 25.56)		No significant difference between groups
	B: Wedge excision (*n* = 53)		24.58 mm (± 28.96)		
Leahy (1990) [40]	A: Chemical ablation (phenol) (*n* = 32)	‘Patient acceptability’ which was partly defined as an absence of severe post-operative pain requiring additional analgesia. 16-month follow-up	One participant found the procedure unacceptably painful		No further analysis is given
	B: Surgical ablation (*n* = 34)		One participant found the procedure unacceptably painful		
Van der Ham (1990) [28]	A: Wedge excision (*n* = 124)	Number of days analgesic was used	68 (54%) participants for a mean number of 1.1 days		*p* < 0.001
	B: Segmental phenol cauterisation (*n* = 125)		25 (20%) participants for 0.4 days		
Issa (1988) [51]	A: Wedge resection (WR) and segmental phenolisation (SP) combination treatment (*n* = 62)	Duration of pain and intensity (No pain, mild, moderate and severe) over 24 h	Duration: 9.4 h [SD 13.5] Intensity: No pain *n* = 19; mild *n* = 20; moderate *n* = 19, severe *n* = 4		Duration: No significant difference was identified between the SP and WR/SP but both groups were significantly shorter than the WR (both *p* < 0.001) Intensity: No significant difference between the SP and WR/SP groups. Both groups were significantly less than the WR group (SP = *p* < 0.001 and WR/SP = *p* < 0.005)
	B: Wedge resection (*n* = 55)		Duration: 30 h [SD 37.6] Intensity: No pain *n* = 5; mild *n* = 8; moderate *n* = 30, severe *n* = 12		
	C: Segmental phenolisation (*n* = 53)		Duration: 6.7 h [SD 13.0] Intensity: No pain *n* = 17; mild n = 18; moderate *n* = 17, severe *n* = 1		
Gerritsma-Bleeker (2002) [46]	A: Partial nail extraction with phenolisation (*n *= 31)	Preoperative, 2 days, 8 days, 1 month, 3 months, 12 months; day- and night-time pain (VAS)	Daytime Pre: 5.9 (2.4) 2 days: 3.8 (2.7) 8 days: 2.3 (1.8) 1 month: 1.6 (1.6) 3 months: 1.2 (0.7) 12 months: 1.7 (1.8)	Night-time Pre: 3.4 (3.0) 2 days: 3.0 (2.7) 8 days: 1.9 (2.2) 1 month: 1.3 (0.9) 3 months: 1.0 (0.2) 12 months: 1.0 (0.0)	Daytime Pre: *p* = 0.980 2 days: *p* = 0.099 8 days: *p* = 0.410 1 month: *p* = 0.160 3 months: *p* = 0.190 12 months: *p* = 0.10 Night-time Pre: *p* = 0.210 2 days: *p* = 0.580 8 days: *p* = 0.240 1 month: *p* = 0.130 3 months: *p* = 0.320 12 months: *p* = 0.360
	B: Partial nail extraction with matrix excision (*n* = 34)		Daytime Pre: 5.9 (2.4) 2 days: 3.8 (2.5) 8 days: 1.9 (1.4) 1 month: 1.2 (0.6) 3 months: 1.8 (2.0) 12 months: 1.2 (0.6)	Night-time Pre: 4.4 (3.1) 2 days: 2.6 (2.2) 8 days: 1.4 (1.3) 1 month: 1.0 (0.2) 3 months: 1.2 (0.8) 12 months: 1.0 (0.2)	
Shaath (2005) [49]	A: Zadik’s procedure (*n* = 38)	1 week (VAS 0–10, 10; being agony)	Not reported		*p* = 0.200
	B: Chemical ablation with Sodium Hydroxide (*n* = 45)		Not reported		
Korkmaz (2013) [38]	A: Partial matrix excision (*n* = 17)	Post-operative pain intensity (mild, moderate, severe). Timepoint unclear	Pain intensity: 3 (17.6%) reported moderate pain. None had severe pain		*P* = > 0.05
	B: Segmental phenolisation (*n* = 22)		Pain intensity: 2 (9%) reported moderate pain. None had severe pain		
Akkus (2018) [18]	A: Chemical matrixectomy with NaOH (*n* = 30)	3 days, 7 days, 1 month after operation (no pain, mild, moderate or severe)	Not reported		Day 3:* p* = 0.001 No significant difference in the pain severity between groups for post-operative Day 7 and Month 1
	B: Wedge resection (*n* = 30		Not reported		
Muriel-Sánchez (2020) [24]	A: Chemical matrixectomy with phenol (*n* = 10)	Post surgical pain at 24, 48 and 72 h (VAS scale 0–10)	24 h: 1.9 ± 1.0 [95% CI 1.31 to 2.49] 48 h:1.2 ± 1.4 [95% CI 0.74 to 1.66] 72 h: 0.8 ± 1.2 [95% CI 0.41 to 1.19]		24 h: *p* = 0.410 48 h: *p* = 0.280 72 h: *p* = 0.330
	B: “Aesthetic reconstruction” (describes partial nail ablation with wedge excision of matrix) (*n* = 24)		24 h: 2.6 ± 2.5 [95% CI 2.04 to 3.16] 48 h: 1.9 ± 2.2 [95% CI 1.41 to 2.39 72 h: 1 ± 1.3 [95% CI 0.71 to 1.29]		
*Chemical v’s Other chemical (n* = *3 studies)*					
Gem (1990) Study 1[21]	A: Chemical ablation with 3-min application of 80% phenol (*n* = 109)	‘days of becoming pain free’	The average time to become pain-free was 3.6 days, with no statistical difference between the groups studied		No statistical analysis information provided
	B: Chemical ablation with 2-min application of 10% sodium hydroxide (*n* = 110)		The average time to become pain-free was 3.6 days, with no statistical difference between the groups studied		
Andre (2018) [33]	A: Nail avulsion and phenol (*n* = 46 toenails)	34 days post-surgery (VAS 0–10)	Overall mean score was below 2/10 for both groups		Pain was initially higher in the trichloroacetic acid group but this decreased faster than in the phenol group. No further information or statistical analysis provided
	B: Nails avulsion and trichloroacetic acid (*n* = 50 toenails)		Overall mean score was below 2/10 for both groups		
Ahsan (2019) [42]	A: Chemical matrixectomy with phenol (*n* = 50)	No pain, mild, moderate, or severe. Timepoint unclear	Severe pain *n* = 2 Moderate pain *n* = 4 Mild pain *n* = 23		*p* = 0.472^*a*^
	B: Chemical matrixectomy with trichloroacetic acid (*n* = 50)		Severe pain *n* = 0 Moderate pain *n* = 4 Mild pain *n* = 19		
*Chemical timings (n* = *3 studies)*					
Gem (1990) Study 2[21]	A: Chemical ablation with 2-min application of 10% sodium hydroxide (*n* = 110)	‘days of becoming pain free’	The average time to become pain-free was 3.6 days, with no statistical difference between the groups studied		No statistical analysis information provided
	B: Chemical ablation with 1-min application of 10% sodium hydroxide (*n* = 93)		The average time to become pain-free was 3.6 days, with no statistical difference between the groups studied		
Tatlican (2009) [27]	A: Partial nail avulsion with 1 min phenol cauterisation (*n* = 37)	2, 10, 16, 24 and 30 days (‘Present’ or ‘absent’)	2 days: 19 (51.4%) Mean days present: 1.4 (± 1.4) days		2 days:* p* = 0.846 Mean Days*: p* = 0.527
	B: Partial nail avulsion with 2-min phenol cauterisation (*n* = 36)		2 days: 16 (44.4%) Mean days present: 1.1 (± 1.2) days		
	C: Partial nail avulsion with 3-min phenol cauterisation (*n* = 37)		2 days: 19 (51.4%) Mean days present: 1.3 (± 1.3) days		
Muriel-Sánchez (2021) [25]	A: Partial nail avulsion with 30 s application of phenol (*n* = 27 halluces [54 nail folds])	24, 48, 72 h post-surgery (VAS)	24 h: 1.7 [SD 0.5] (95% CI 1.5–1.9; Median 2, IQR 1) 48 h: 1.9 [SD 1.8] (95% CI 1.19–2.61; Median 1, IQR 2) 72 h: 1.2 [SD 1.3] (95% CI 0.69–1.71; Median 1, IQR 2)		24 h: *p* = 0.650 48 h: *p* = 0.720 72 h: *p* = 0.790
	B: Partial nail avulsion with 60 s application of phenol (*n* = 27 halluces [54 nail folds])		24 h: 1.6 [SD 0.6] (95% CI 1.6–1.84; Median 2, IQR 1) 48 h: 1.1 [SD 1.3] (95% CI 0.59–1.61; Median 1, IQR 2) 72 h: 0.7 [SD 1.1] (95% CI 0.26–1.14; Median 0, IQR 1)		
*Chemical matrixectomy v’s Surgical* + *chemical matrixectomy (n* = *2 studies)*					
Issa (1988) [51]	A: Wedge resection and segmental phenolisation combination treatment (*n* = 62)	Duration of pain and intensity (No pain, mild, moderate and severe) over 24 h	Duration: 9.4 h [SD 13.5] Intensity: No pain *n* = 19; mild *n* = 20; moderate *n* = 19, severe *n* = 4		Duration: No significant difference was identified between the SP and WR/SP but both groups were significantly shorter than the WR (both *p* < 0.001) Intensity: No significant difference between the SP and WR/SP groups. Both groups were significantly less than the WR group (SP = *p* < 0.001 and WR/SP = *p* < 0.005)
	B: Wedge resection (*n* = 55)		Duration: 30 h [SD 37.6] Intensity: No pain *n* = 5; mild *n* = 8; moderate *n* = 30, severe *n* = 12		
	C: Segmental phenolisation (*n* = 53)		Duration: 6.7 h [SD 13.0] Intensity: No pain *n* = 17; mild *n* = 18; moderate *n* = 17, severe *n* = 1		
Alvarez-Jimenez (2011) [31]	A: Phenol and curettage (*n* = 73 nail folds)	Post-operative pain 2 days after procedure (10 cm VAS scale, 0 no pain,10 maximum pain)	3.95 ± 2.25		*p* = 0.028
	B: Phenol (*n* = 79 nail folds)		3.06 ± 2.21		
*Chemical matrixectomy v’s ‘Other’ (e.g., laser and electrocautery) (n* = *1 study)*					
Hamid (2021) [36]	A: Partial nail avulsion and matrixectomy with phenol (*n* = 50)	Post-operative pain (mild, moderate or severe). Timepoint unclear	Mild pain (*n* = 17/50) Moderate pain (*n* = 6/50) Severe pain (*n* = 1/50)		Mild: *p* = 0.660 Moderate: *p* = 1.00 Severe:* p* = 1.00
	B: Partial nail avulsion and matrixectomy with electrocautery (*n* = 50)		Mild pain (*n* = 14/50) Moderate pain (*n* = 6/50) Severe pain (*n* = 2/50)		
*Surgical matrixectomy v’s Surgical* + *chemical matrixectomy (n* = *1 study)*					
Issa (1988) [51]	A: Wedge resection and segmental phenolisation combination treatment (*n* = 62)	Duration of pain and intensity (No pain, mild, moderate and severe) over 24 h	Duration: 9.4 h [SD 13.5] Intensity: No pain *n* = 19; mild *n* = 20; moderate *n* = 19, severe *n* = 4		Duration: No significant difference was identified between the SP and WR/SP but both groups were significantly shorter than the WR (both *p* < 0.001) Intensity: No significant difference between the SP and WR/SP groups. Both groups were significantly less than the WR group (SP = *p* < 0.001 and WR/SP = *p* < 0.005)
	B: Wedge resection (*n* = 55)		Duration: 30 h [SD 37.6] Intensity: No pain *n* = 5; mild *n* = 8; moderate *n* = 30, severe *n* = 12		
	C: Segmental phenolisation (*n* = 53)		Duration: 6.7 h [SD 13.0] Intensity: No pain *n* = 17; mild *n* = 18; moderate *n* = 17, severe *n* = 1		
*Surgical matrixectomy v’s ‘Other’ (e.g., laser and electrocautery) (n* = *2 studies)*					
Kavoussi (2020) [22]	A: Partial Nail Matrixectomy using CO_2_ laser (*n* = 62)	Duration of pain. Timepoint unclear. Participants were followed over 24 months	3.20 days [± 1.734]		*p* = 0.620
	B: Lateral Nail Fold Excision (LNFE) (*n* = 65)		3.66 days [± 2.111]		
Awad (2020) [20]	A: Partial nail matrixectomy with electrocautery (*n* = 100)	Day 3 and 7 (Likert Scale, none, mild moderate or severe pain)	Three days: 32% no pain, 15.5% mild, 2.5% moderate, 0% severe Seven days: 44.5% no pain, 3% mild, 2.5% moderate, 0% severe		*p* = 0.018 +
	B: Partial nail matrixectomy (*n* = 100)		Three days: 39.5% no pain, 9% mild, 1.5% moderate, 0% severe Seven days: 46% no pain, 2.5% mild, 1.5% moderate, 0% severe		
*Surgical v’s Surgical (n* = *1 study)*					
Habeeb (2020) [47]	A: Central toenail resection (*n* = 50)	2, 3, 4 days post-operative pain (absent or present)	Day 2: present in 12 participants Day 3: present in 2 participants Day 4: present in 0 participants		Day 2: *p* < 0.001 Day 3: *p* = 0.004 Day 4: *p* = 0.001
	B: Wedge toenail resection (*n* = 50)		Day 2: present in 35 participants Day 3: present in 12 participants Day 4: present in 10 participants		
*Chemical matrixectomy v’s Avulsion only (n* = *1 study)*					
Khan (2014) [37]	A: Partial Nail Avulsion + Phenol (*n* = 50)	Day 3 and 7 post-operative pain (none, mild, moderate and severe)	Day 3: 2% no pain, 31% mild, 12% moderate, 5% severe Day 7: 40% no pain, 6% mild, 3% moderate, 1% severe		*p* = 0.018^*a*^
	B: Partial Nail Avulsion alone (*n* = 50)		Day 3: 0% no pain, 18% mild, 22% moderate, 10% severe Day 7: 35% no pain, 10% mild, 3% moderate, 2% severe		
*Anaesthetics (with and without epinephrine) (n* = *2 studies)*					
Altinyazar (2010) [30]	A: Plain lidocaine (*n* = 22)	1-day post-operative pain (mild, moderate, severe)	Mild pain *n* = 10 Moderate pain *n* = 2 Severe pain *n* = 0		There was no statistically significant difference in postoperative pain
	B: Lidocaine with epinephrine (*n* = 22)		Mild pain *n* = 9 Moderate pain *n* = 2 Severe pain *n* = 0		
Cordoba-Fernandez (2015) [43]	A: Segmental phenolisation matrixectomy with anaesthetic digital block with epinephrine (*n* = 34 toes)	3 days post-surgery (Scale 1–10)	Day 1: 4 (2.74) Day 2: 4.07 (2.26) Day 3: 3.24 (1.73)		*p* = > 0.05^*a*^
	B: Segmental phenolisation matrixectomy with anaesthetic digital block without epinephrine (36 toes)		Day 1: 3.92 (1.85) Day 2: 4.64 (1.98) Day 3: 2.94 (1.98)		

*SD* Standard Deviation, *CI* Confidence Interval, *OR* Odds Ratio, *NaOH* Sodium Hydroxide

^*^Unless otherwise specified

^*a*^Unclear on the timepoints included in the analysis

### Participant satisfaction

Participant satisfaction was reported in nine studies [20, 21, 33, 36, 40–42, 47, 53] (Table 5). All studies reported improvements in satisfaction, although how that was defined and measured was generally unclear. Two studies [40, 47] measured this using a VAS of 0–10 and only three studies [40, 47, 51] undertook statistical analysis. ‘Satisfaction with scar’ was found to be higher with chemical matrixectomy than matrix excision [47] at 1 month but this difference was no longer significant by 3 and 12 months. The same study also measured ‘satisfaction with cosmetic results’ and found no significant difference at any of the timepoints. In a comparison of nail bracing to matrix excision [40], satisfaction was higher in the matrix excision group at 4 and 26 weeks.

**Table 5:** Outcome measure: Participant Satisfaction (*n* = 9)

**Author (Year)**	**Interventions**	**Timepoint**	**Satisfaction** (mean ± SD)*	**Significance** (*p* value)
*Conservative treatment (e.g., braces and gutter treatment) v’s Chemical matrixectomy (n* = *2 studies)*				
AlGhamdi (2014) [19]	A: Lateral nail avulsion with phenol (*n* = 30)	Timepoint unclear	Both groups were equally satisfied with their treatment	No statistical analysis undertaken
	B: Nail tube splinting (*n* = 23)		Both groups were equally satisfied with their treatment	
Ceren (2013) [50]	A: Partial nail extraction with phenol matrixectomy (*n* = 63 toenails)	Pre-operative vs post-operative at 2 days, 1 and 6 months post procedure	Not reported	Cosmetic satisfaction scores were greater than preoperative scores on the second day and at 1 and 6 months in both groups (p < .001). These scores did not differ significantly between the two groups
	B: Partial nail elevation and flexible tube (57 toenails)		Not reported	
*Conservative treatment (e.g., braces and gutter treatment) v’s surgical matrixectomy (n* = *2 studies)*				
Kruijff (2008) [45]	A: Partial nail extraction with partial matrix excision (*n* = 58)	4 weeks, 26 weeks and 12 months (1–10, 10 very satisfied)	4 weeks: 7.3 (median) 26 weeks: 8.74 (median) 12 months: Not reported	4 weeks: *p* < 0.040 26 weeks: *p* = 0.001 12 months: No significance difference stated
	B: Orthonyxia (*n* = 51)		4 weeks: 8.43 (median) 26 weeks: 9.57 (median) 12 months: Not reported	
Peyvandi (2011) [41]	A: Winograd method (*n* = 50)	6 months	Not reported	The majority of patients were satisfied more with the sleeve than the Winograd method. No further information provided
	B: Sleeve (gutter) method (*n* = 50)		Not reported	
*Chemical matrixectomy v’s Surgical matrixectomy (n* = *2 studies)*				
Leahy (1990) [40]	A: Chemical ablation (phenol) (*n* = 32)	Assessed as ‘good’ or ‘poor’ between 16 and 30 months	Good: *n* = 19/32 Poor: *n* = 13/32	No statistical analysis undertaken
	B: Surgical ablation (*n* = 34)		Good: *n* = 22/34 Poor: *n* = 12/32	
Gerritsma-Bleeker (2002) [46]	A: Partial nail extraction with phenolisation (*n* = 31)	1, 3 and 12 months; satisfaction with scar’ and ‘satisfaction with cosmetic result’	Satisfaction with scar: 1 month: 2.1 ± 2.2 3 months: 1.3 ± 1.0 12 months: 1.7 ± 2.2 Satisfaction with cosmetic result: 1 month: 1.1 ± 2.1 3 months: 1.0 ± 2.1 12 months: 2.0 ± 3.0	Satisfaction with scar: 1 month: *p* = 0.020 3 months: *p* = 0.370 12 months: *p* = 0.460 Satisfaction with cosmetic result: 1 month: *p* = 0.550 3 months: *p* = 0.110 12 months: *p* = 0.170
	B: Partial nail extraction with matrix excision (*n* = 34)		Satisfaction with scar: 1 month: 1.2 ± 0.4 3 months: 1.7 ± 1.8 12 months: 1.3 ± 1.2 Satisfaction with cosmetic result: 1 month: 1.4 ± 2.7 3 months: 2.2 ± 3.2 12 months: 1.0 ± 1.9	
*Chemical matrixectomy v’s Surgical* + *chemical matrixectomy (n* = *1 study)*				
Anderson (1990) [32]	A: Nail bed excision (*n* = 17)	Timepoint unclear	1 participant expressed dissatisfaction	No statistical analysis undertaken
	B: Combination of nail bed phenolisation and excision (*n* = 14)		No reports of dissatisfaction	
*Chemical matrixectomy v’s ‘Other’ (e.g., laser and electrocautery) (n* = *1 study)*				
Awad (2020) [20]	A: Partial nail matrixectomy with electrocautery (*n* = 100)	Aesthetic results^*a*^ after 1 and 6 months	Good: 97 (48.5%)	No statistical analysis undertaken
	B: Partial nail matrixectomy (*n* = 100)		Good: 99 (49.5)	
*Chemical matrixectomy v’s Avulsion only (n* = *1 study)*				
Greig (1991) [35]	A: Total avulsion (*n* = 81 nail edges)	Timepoint unclear	Satisfied: 27 participants of 59 (46%)	No statistical analysis undertaken
	B: Nail edge excision (*n* = 56 nail edges)		Satisfied: 23 participants of 47 (49%)	
	C: Nail edge excision and phenolisation (*n* = 67 nail edges)		Satisfied: 48 participants of 57 (84%)	

*SD* Standard Deviation, *CI* Confidence Interval*, OR* Odds Ratio, *NaOH* Sodium Hydroxide

^*^Unless otherwise specified

^*a*^Unclear on the timepoints included in the analysis

### Risk of bias

**Fig. 1:**
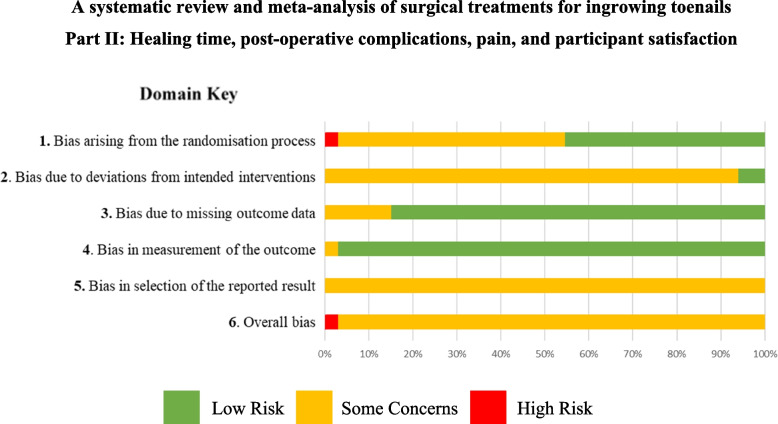
Risk of bias summary plot: RoB 2.0 tool

We used the used the Cochrane RoB 2.0 tool and assessed six domains for each study. No study was rated as low risk, for reasons such as not or providing information surrounding the randomisation process, not including all participants in the final analysis and failing to provide information on blinding of participants or the outcome assessor. Risk of bias summaries are presented in Fig. 1 and risk of bias table in Supplementary Table 2.

## Discussion

This is the second paper from this systematic review and meta-analysis of randomised controlled rials of surgical treatments for ingrown toenails. The first reported the methods used in the review and reported results from the primary outcomes of recurrence and relief of symptoms [15]. This second paper focusses on the secondary outcomes of healing time, postoperative complications (e.g., infection and haemorrhage), postoperative pain (duration and intensity) and participant satisfaction. Although a large number of trials were identified for inclusion in the review, the poor reporting, heterogeneity of the studies and differences in outcome measures/timepoints, meant a meta-analysis was not possible on these secondary outcomes.

Perhaps the most obvious clinical finding from this, is the lack of robust clinical conclusions that can be drawn from all these studies. Possibly the clearest pattern to emerge was around the use of phenol. Shorter application of phenol during the chemical matrixectomy was linked to shorter time to healing in two studies, but application duration appeared to have little effect on post operative complications [26, 28]. Although this may suggest that clinicians should use a shorter duration of phenol, this must be balanced against the meta-analysis in the first paper which indicate higher rates of regrowth are associated with shorter application times[15]. The optimal balance of effectiveness and sequalae is yet to be determined and clinicians may opt to vary application times to meet the needs of individual patients.

Curettage has been explored in several studies. Alvarez-Jimenez et al. reported that using a Martini bone curette following partial nail avulsion and destruction of the nail matrix with phenol reduced healing time by a third (7.5 ± 1.8 days compared to 12.4 ± 3 days, *p* = 0.001) [32]. They also found that it reduced rates of post operative infection, but increased post operative bleeding and as reported previously had no effect on recurrence[15]. However, with only 51 patients, and that this has not been tested in multiple trials, care must be taken not to overinterpret these findings. It is notable that whilst interventions such as curettage may benefit some outcomes such as healing, it may increase others such as post operative bleeding. A similar pattern was found with phenol where longer durations of application were linked to reduced likelihood of regrowth but increase healing times [15, 26, 28]. Clinicians and future studies should prioritise these competing risks and benefits in a way that prioritises what is important to patients.

Many studies report infection rates following nail surgery but combining these isn’t yet possible as case definitions are unclear and inconsistent. Standardised definitions of surgical site infections, and severity classifications exist and have been used in other fields of surgical research, but they have not yet been validated and applied to nail surgery [54–56]. Despite the clear interest in post operative infection as an outcome, only one trial explored the use of oral antibiotics and found no evidence that they reduced the rate of post operative infection. However, with only 50 to 53 participants per group, it would only have been powered to identify a large effect.

Other post operative sequalae, such as haemorrhage also had unclear case definitions and were poorly reported. With some studies only capturing data for some outcomes up to 48 h post procedure [32], there is not enough time to meaningfully assess the effect of an intervention on complication rates. Perhaps more worryingly, there was a lack of information on the reporting of adverse events in general despite clear legal and governance frameworks being in place for many years.

Another frequently captured outcome was patient satisfaction. This is a widely used, but poorly defined concept in healthcare and although definitions vary, they generally centre on satisfaction being the extent to which an individual’s experience meets their expectations [57–59]. However, patient expectations are not a stable trait and change over time as has been recognised elsewhere [60]. Evidence from randomised trials have shown that patient expectations can be deliberately modified, and that patient expectations can be guided towards what clinicians consider achievable [60, 61]. Modification of patient’s expectations would in turn influence their final level of satisfaction, which brings into question its value as a measure of treatment effectiveness.

Given the limitations of the studies identified in this review, it’s clear that many fundamental questions remain unanswered around the surgical treatment of ingrown toenails: Is destruction of the nail matrix always necessary? What is the optimal technique to prevent symptomatic regrowth? How should patients be reviewed and monitored post-operatively? Are different procedures more appropriate for subgroups of patients? Further high-quality collaborative trials are needed to answer these questions.

Findings from this paper should be interpreted in line with the assessments of risk of bias and certainty of evidence reported in the first paper [15]. All studies included in the review were assessed as having either high risk or having some concerns about bias when assessed with the Cochrane ROB 2.0 tool. Similarly, none of the comparisons were considered to have high certainty when assessed with the GRADE system in the first paper [15]. These issues could have been averted at the protocol development stage of each trial and there is a large body of literature to guide the development and conduct of such trials [62–66]. Similarly, frameworks exist that would aid reporting and peer review of such studies [67, 68]. This is a clear case of what the late Prof Doug Altman, who perhaps did more to improve healthcare research than anyone else, referred to when he said “We need less research, better research, and research done for the right reasons” [69].

## Conclusion

This paper reports the narrative synthesis of the secondary outcomes from a systematic review and meta-analysis of randomised trials on surgical treatments for ingrown toenails. Despite the large volume of trials published in this area, poor design and reporting of studies prevented meta-analysis of these outcomes and limits the clinical conclusions that can be drawn. What is clear is that further robust, patient centred, clinical trials are urgently needed to fill the vacuum of quality evidence around such a commonly performed procedure.

### Supplementary Information

**Additional file 1. **Full search strategy. 

**Additional file 2. **PRISMA flow diagram of literature search and study selection phases; n, number; WoS, Web Of Science; CENTRAL, Cochrane Central Register of Controlled Trials; WHO ICTRP, World Health Organisation International Clinical Trials Registry Platform; ISRCTN, International Standard Randomised Controlled Trial Number Registry.

**Additional file 3: Supplementary Table 1.** Characteristics of included studies.

**Additional file 4: Supplementary Table 2.** Risk of bias summary table.

## Data Availability

All data are available from the corresponding author on reasonable request.

## Abbreviations

CENTRAL: Cochrane Central Register of Controlled Trials
CI: Confidence Interval
GRADE: Grades of Research, Assessment, Development and Evaluation
ISRCTN: International Clinical Trials Registry
MD: Mean Differences
MESH: Medical Subject Heading
PRISMA: Preferred Reporting Items for Systematic Reviews and Meta-Analyses
PROSPERO: International Prospective Register of Systematic Reviews
RCT: Randomised Controlled Trial
ROB: Risk of Bias
RR: Risk Ratio
VAS: Visual Analogue Scale
