# Supplementary material for: A systematic review and meta-analysis of randomised controlled trials of surgical treatments for ingrown toenails part II: healing time, post-operative complications, pain, and participant satisfaction
Source: J Foot Ankle Res. 2023 Sep 6;16:55. doi: 10.1186/s13047-023-00655-7 (PMC10481456; doi:10.1186/s13047-023-00655-7)
Supplement: Supplementary file 2 — Additional file 2. PRISMA flow diagram of literature search and study selection phases; n, number; WoS, Web Of Science; CENTRAL, Cochrane Central Register of Controlled Trials; WHO ICTRP, World Health Organisation International Clinical Trials Registry Platform; ISRCTN, International Standard Randomised Controlled Trial Number Registry. [file 13047_2023_655_MOESM2_ESM.docx]

Records Screened:
(**n = 1641**)

Records sought for retrieval and assessed for eligibility:

(**n = 0**)

Records identified from:
**Database (n = 3799)**
[*MEDLINE* n = 621, *EMBASE* n = 1479, *CINAHL* n = 120, WoS n = 1203, *CENTRAL* n = 247]

**Registers** (**n = 129**)
[*Clinicaltrials* n = 129, *ISRCTN* n = 0]

## Identification

**Identification of studies via databases and registers**

**Identification of studies via other methods**

Records identified from:
**Citation Tracking (n = 0)**

Reasons removed before screening:

Duplicates: (**n =2287**)

Records Excluded:
(**n = 1574**)

Reports sought for retrieval and assessed for eligibility:
(**n = 70**)

## Screening

Full-text articles excluded, with reasons: (**n = 34**)

- Not an RCT/ Wrong study design n=18
- Not in English n= 2
- Wrong outcome n=1
- Quasi randomised n= 9
- Trial registration n= 3
- Not enough information (abstract only) n=1

Studies included in qualitative synthesis*: (**n = 36**)

Studies included in quantitative synthesis [meta-analysis]: (**n = 31**)

## Included

Full-text articles excluded, with reasons: (**n = 0**)

**Supplementary File 2**. PRISMA flow diagram of literature search and study selection phases; n, number; WoS, Web Of Science; CENTRAL, Cochrane Central Register of Controlled Trials; WHO ICTRP, World Health Organisation International Clinical Trials Registry Platform; ISRCTN, International Standard Randomised Controlled Trial Number Registry
