# Supplementary material for: A systematic review and meta-analysis of randomised controlled trials of surgical treatments for ingrown toenails part II: healing time, post-operative complications, pain, and participant satisfaction
Source: J Foot Ankle Res. 2023 Sep 6;16:55. doi: 10.1186/s13047-023-00655-7 (PMC10481456; doi:10.1186/s13047-023-00655-7)
Supplement: Supplementary file 3 — Additional file 3: Supplementary Table 1. Characteristics of included studies. [file 13047_2023_655_MOESM3_ESM.docx]

| **Supplementary Table 1.** Characteristics of included studies | | | | | |
| --- | --- | --- | --- | --- | --- |
| **Author (Year)** | **Study Design (Country)** | **Follow-Up** | **Participants** | **Interventions** | **Outcome Measures** |
| Ahsan (2019) [48] | RCT (Pakistan) | 3 months | 100 participants; Mean age 26.31 + 8.74; 45/46 M/F; Severity not reported | A: Chemical matrixectomy with phenol (n=50) | 1. Healing time at 48 hours, week 1, 4 and 12 up to 3 months. 2. Wound infection and discharge 3. Pain (no pain, mild, moderate, severe) 4. Recurrence up to 3 months |
|  |  |  |  | B: Chemical matrixectomy with trichloroacetic acid (n=50) |  |
| Akkus (2018) [31] | RCT (Turkey) | 12 months | 60 participants (75 procedures); Mean age 30 – 31.7; 27/23 M/F; Stage I (n=32), Stage II (n=19), Stage III (n=24) | A: Chemical matrixectomy with NaOH (n=30) | 1. Recurrences at 12 months 2. Healing time assessed at day 3, Week 1, Month 1, 6, and 12 3. Pain (pain severity- mild, moderate or severe) 4. Operation time 5. Post-operative drainage 6. QOL (Dermatologic Life Quality Index) |
|  |  |  |  | B: Wedge resection (n=30) |  |
| AlGhamdi (2014) [28] | RCT (Saudi Arabia) | 6 months | 53 participants; Mean age 47.7 + 1.3 (range 10-60 years); 46/7 M/F; Stage II and III | A: Lateral nail avulsion with phenol (n=30) | 1. Symptomatic regrowth 1, 3 and 6 months 2. Healing time 1, 3 and 6 months 3. Significant complications including infection 4. Pain at baseline, 1, 3 and 6 months 5. Participant satisfaction 6. Drainage 7. Shoe-wear discomfort 8. Overall success rate |
|  |  |  |  | B: Nail tube splinting (n=23) |  |
| Altinyazar (2010) [55] | RCT (Turkey) | 18 months | 44 participants; Mean 28.5-32.8; 21/23 M/F; All stage III | A: Plain lidocaine (n=22) | 1. Recurrence at 18 months 2. Healing time 3. Pain 4. Drainage |
|  |  |  |  | B: Lidocaine with epinephrine (n=22) |  |
| Alvarez-Jimenez (2011) [43] | RCT; Double- Blinded (Spain) | 12 months | 51 participants (152 procedures); Mean age 34.05 + 19.23; 18/33 M/F; Stage I or IIa | A: Phenol and curettage (n=73 nail folds) | 1. Recurrence at 12 months 2. Healing time over 1 months (digital photo) 3. Infection 4. Bleeding over 48 hours 5. Pain 2 days post-surgery (VAS) |
|  |  |  |  | B: Phenol (n= 79 nail folds) |  |
| Anderson (1990) [45] | RCT (UK) | 12 months | 31 participants; Age range 15-73 years); 19/11 M/F; Severity not reported | A: Nail bed excision (n=17) | 1. Recurrence at 12 months 2. Post operative infection at 2 weeks 3. Participant satisfaction at 2 weeks and 12 months 4. Previous operations and relation to recurrence |
|  |  |  |  | B: Combination of nail bed phenolisation and excision (n=14) |  |
| Andre (2018) [46] | RCT; Double- Blinded (France) | 4 months | 84 participants (96 toenails); Age range 14-88 years; 41/40 M/F; Severity not reported | A: Nail avulsion and phenol (n=46 toenails) | 1. Recurrence at 4 months 2. Inflammation at day 2, week 2, 4 and month 4 3. Pain at 34 days post-surgery (VAS) 4. Presence of oozing 5. Postoperative morbidity |
|  |  |  |  | B: Nails avulsion and trichloroacetic acid (n=50 toenials) |  |
| Awad (2020) [51] | RCT (Saudi Arabia) | 6 months | 200 participants; Mean age 26.45 (range 16-52 years); 136/64 M/F; Stage I (n=66), Stage II (n=38), Stage III (n=96) | A: Partial nail matrixectomy with electrocautery (n=100) | 1. Recurrence at 1 and 6 months 2. Healing time assessed at 3 and 7 days, 1 and 6 months 3. Infection assessed at 3 and 7 days 4. Pain assessed at day 3 and 7 (VAS) 5. Participant satisfaction at 1 and 6 months |
|  |  |  |  | B: Partial nail matrixectomy (n=100) |  |
| Bos (2007) [32] | RCT (Netherlands) | 12 months | 123 participants; Age range 9-73; 72/45 M/F; Severity not reported | A: Partial avulsion with excision of the matrix (n=38) | 1. Recurrence at 1, 6 and 12 months 2. Infection at 2 days, 1 week and 1 month |
|  |  |  |  | B: Partial avulsion of the matrix and application of antibiotic (n=22) |  |
|  |  |  |  | C: Partial avulsion with application of phenol (n=37) |  |
|  |  |  |  | D: Partial avulsion with application of phenol and antibiotic (n=26) |  |
| Ceren (2013) [29] | RCT (Turkey) | 6 months | 120 toenails; Age range (11-65 years); 59/48 M/F; Stage I (n=31), Stage II (n=17), Stage III (n=72) | A: Partial nail extraction with phenol matrixectomy (n=63 toenails) | 1. Recurrence at 6 months 2. Haemorrhage or discharge at 6 months 3. Pain at 6 months 4. Cosmetic satisfaction at 6 months 5. Time to recovery |
|  |  |  |  | B: Partial nail elevation and flexible tube (57 toenails) |  |
| Cordoba-Fernandez (2015) [54] | RCT (Spain) | 12 months | 44 participants (10 toes); Mean age 26.28 + 15.82 (range 10-64 years); 21/23 M/F; Severity not reported | A: Segmental phenolisation matrixectomy with anaesthetic digital block with epinephrine (n=34 toes) | 1. Recurrence 2. Bleeding 3. Pain at 1-, 2- and 3-days post-op (VAS) 4. Duration of anaesthetic effect |
|  |  |  |  | B: Segmental phenolisation matrixectomy with anaesthetic digital block without epinephrine (36 toes) |  |
| Gem (1990) Study 1[22] | Prospective RCT (UK) | 18 months | 219 participants; age, gender and severity not reported | A: Chemical ablation with 3-minute application of 80% phenol (n=109) | 1. Recurrence at 18 months 2. Time to become pain free 3. Relief of symptoms 4. Healing time |
|  |  |  |  | B: Chemical ablation with 2-minute application of 10% sodium hydroxide (n=110) |  |
| Gem (1990) Study 2 [23] | Prospective RCT (UK) | 18 months | 203 participants; age, gender and severity not reported | A: Chemical ablation with 2-minute application of 10% sodium hydroxide (n=110) | 1. Recurrence at 18 months 2. Time to become pain free 3. Relief of symptoms 4. Healing time |
|  |  |  |  | B: Chemical ablation with 1-minute application of 10% sodium hydroxide (n=93) |  |
| Gerritsma-Bleeker (2002) [33] | RCT (Netherlands) | 12 months | 60 participants (63 procedures); Mean age 22.7- 24.4; 30/33 M/F; Severity not reported | A: Partial nail extraction with phenolisation (n=31) | 1. Relief of symptoms at 1, 3 and 12 months (VAS) 2. Recurrence at 1, 3 and 12 months 3. Pain at 2 days, 8 days, 1 month, 3 months, 12 months (VAS) 4. Participant satisfaction with scar and cosmetic result at 1, 3 and 12 months (VAS) 5. Erythema & purulent exudates 6. Morbidity 7. Time to complete recovery |
|  |  |  |  | B: Partial nail extraction with matrix excision (n=34) |  |
| Greig (1991) [42] | RCT (Scotland) | 12 months | 163 participants (204 procedures); Age range 12-77 years; 113/50 M/F; Severity not reported | A: Total avulsion (n=81 nail edges) | 1. Recurrences at 12 months 2. Postoperative infection after 2 weeks 3. Participant satisfaction at 12 months |
|  |  |  |  | B: Nail edge excision (n=56 nail edges) |  |
|  |  |  |  | C: Nail edge excision and phenolisation (n=67 nail edges) |  |
| Habeeb (2020) [44] | RCT (Egypt) | 6 months | 100 participants; Age range 10-35 years; 78/22 M/F; Severity not reported | A: Central toenail resection (n=50) | 1. Relief of symptoms at 4 and 8 weeks 2. Recurrences at 6 months 3. Pain at 1,2,3 and 4 days post-op 4. Duration of technique |
|  |  |  |  | B: Wedge toenail resection (n=50) |  |
| Hamid (2021) [25] | RCT (Pakistan) | 6 months | 100 participants; Mean age 19.7-20.2; gender and severity note reported | A: Partial nail avulsion and matrixectomy with phenol (n=50) | 1. Recurrence at 3 and 6 months 2. Serous and Purulent discharge at 7th and 14th days and at week 4 and 6 3. Pain |
|  |  |  |  | B: Partial nail avulsion and matrixectomy with electrocautery (n=50) |  |
| Issa (1998) [34] | RCT (Ireland) | 6 months | 140 participants (170 procedures); Mean age 21 (range 9-54 years); 65/22 M/F; Severity not reported | A: Wedge resection and segmental phenolisation combination treatment (n=62) | 1. Recurrence at 6 months 2. Pain duration at 1-6 hrs, 6-12 hours, 12-24 hours, >24 hours post-op 3. Pain intensity using the linear pain analogue scale |
|  |  |  |  | B: Wedge resection (n=55) |  |
|  |  |  |  | C: Segmental phenolisation (n=53) |  |
| Kavoussi (2020) [50] | RCT (Iran) | 24 months | 127 participants; Mean age 28.2-28.9; 68/59 M/F; Stage I (n=7), Stage IIa (n=40), Stage IIb (n=51), Stage III (n=21), Stage IV (n=8) | A: Partial Nail Matrixectomy using CO₂ laser (n=62) | 1. Spicule formation 2. Healing time 3. Infection 4. Pain 5. Cosmetic outcome 6. Time to return to daily activity 7. Time to return to work |
|  |  |  |  | B: Lateral Nail Fold Excision (LNFE) (n=65) |  |
| Khan (2014) [41] | RCT (Pakinstan) | 6 months | 100 participants; Mean age 18 (range 14-45 years); M:F ratio= 2.3:1; Severity not reported | A: Partial Nail Avulsion + Phenol (n=50) | 1. Recurrence or spike formation at 1 and 6 months 2. Infection at 3^rd^ and 7^th^ day post-op 3. Pain at 3^rd^ and 7^th^ day post-op (VAS) 4. Mortality |
|  |  |  |  | B: Partial Nail Avulsion alone (n=50) |  |
| Kim (2015) [52] | RCT (Korea) | 6 months | 61 participants; Mean age 20.0 – 20.3; All male; Grade 2 or 3 | A: Curettage (n=32) | 1. Recurrences at 6 months 2. Infection at 3-5 days post-op |
|  |  |  |  | B: Electrocautery (n=29) |  |
| Korkmaz (2013) [35] | RCT (Turkey) | Mean 3.2 ± 1.2 years | 39 participants; Mean age 16.1 – 17.0; 28/11 M/F; Stage 2 (n=13) or 3 (n=26) | A: Partial matrix excision (n=17) | 1. Recurrence 2. Infection 3. Pain (Duration of analgesic usage) 4. Return to work time |
|  |  | Mean 2.1 ± 0.9 years |  | B: Segmental phenolisation (n=22) |  |
| Kruijff (2008) [27] | RCT (Netherlands) | 12 months | 105 participants (109 nails procedures); Mean age 25.3 + 15.2; 73/36 M/F; Severity not reported | A: Partial nail extraction with partial matrix excision (n=58) | 1. Recurrence at 12 months 2. Redness, exudate and post-operative bleeding at 1 week 3. Pain at 4, 12 and 26 weeks (VAS) 4. Participant satisfaction at 4 and 26 weeks (VAS) 5. Time to complete recovery 6. Relief of symptoms (VAS) |
|  |  |  |  | B: Orthonyxia (n=51) |  |
| Leahy (1990) [36] | RCT (Ireland) | 30 months | 66 participants; Mean age 24 years; 48% females; Severity not reported | A: Chemical ablation (phenol) (n=32) | 1. Spicules or spikes regrowth at 3 months and between 16 and 30 months 2. Infection at 1 week, 3 months, and between 16 and 30 months 3. Haemorrhage at 1 week, 3 months, and between 16 and 30 months 4. Cosmetic outcome between 16 and 30 months 5. Postoperative pain (absence of pain relief) at 1 week, 3 months and between 16 and 30 months 6. Participant satisfaction at 16 and 30 months |
|  |  |  |  | B: Surgical ablation (n=34) |  |
| Misiak (2014) [53] | RCT (Poland) | 3 months | 60 participants; Mean age 41.4 + 9.95 (range 26-64 years); 32/28 M/F; Grade 3 and 4 | A: Partial nail extraction + phenolisation (n=30) | 1. Recurrence at 1, 2 and 3 months 2. Healing time at 7 days, 14 days, 1, 2 and 3 months |
|  |  |  |  | B: Partial nail extraction + electrocautery (n=30) |  |
| Morkane (1984) [37] | RCT (New Zealand) | 14 months | 103 participants (107 procedures); Mean age 24.6 – 28.5; M/F ratio 3.5:1; Severity not reported | A: Segmental or angular phenolisation (n=54) | 1. Regrowth at mean follow up time of 14 months 2. Pain measured 1 week post-operatively (linear analogue scale) |
|  |  |  |  | B: Wedge excision (n=53) |  |
| Muriel-Sánchez (2020) [38] | RCT (Spain) | 6 months | 34 participants (112 procedures); Mean age 34 + 18.3; 12/22 M/F; Stage I or IIa | A: Chemical matrixectomy with phenol (n=10) | 1. Recurrence at 6 months 2. Healing time 3. Post-surgical bleeding at first dressing 4. Infection 5. Pain at 24, 48, 72 hours (VAS) 6. Post-surgical inflammation |
|  |  |  |  | B: “Aesthetic reconstruction” (describes partial nail ablation with wedge excision of matrix) (n=24) |  |
| Muriel-Sánchez (2021) [49] | RCT (Spain) | 6 months | 27 participants (108 procedures); Mean age 36 + 10.7 years; 8/19 M/F; Stages I or IIa | A: Partial nail avulsion with 30 second application of phenol (n= 27 halluces [54 nail folds]) | 1. Recurrence at a minimum of 6 months 2. Healing time 3. Inflammation, bleeding & infection at 72 hours then twice weekly until healed 4. Pain at 1, 2 and 3 days post-op (VAS) |
|  |  |  |  | B: Partial nail avulsion with 60 second application of phenol (n= 27 halluces [54 nail folds]) |  |
| Peyvandi (2011) [30] | Prospective RCT (Iran) | 6 months | 100 participants; Mean age 27.8 (range 12-47 years); 54/46 M/F; Severity not reported | A: Winograd method (n=50) | 1. Recurrence at 1 week, 1 and 6 months 2. Infection at 1 week, 1 and 6 months 3. Postoperative workday loss 4. Surgery duration 5. Participant satisfaction |
|  |  |  |  | B: Sleeve (gutter) method (n=50) |  |
| Reyzelman (2000) [21] | RCT (US) | Until healed | 154 participants; Mean age 20.7 + 8.6 (range 10-60 years); 91/63 M/F; Severity not reported | A: 1 week course of oral antibiotics and simultaneous phenol matrixectomy (n=53) | 1. Healing time assessed at 3-4 days then weekly until healing occurred 2. Infection assessed at 3-4 days then weekly until healing occurred |
|  |  |  |  | B: 1 week course of oral antibiotics and phenol matrixectomy 1 week later (n=51) |  |
|  |  |  |  | C: Phenol matrixectomy without antibiotic therapy (n=50) |  |
| Shaath (2005) [40] | RCT (UK) | 12 months | 83 participants; Mean age 37.2 – 39.4 years; 53/30 M/F; Severity not reported | A: Zadik’s procedure (n=38) | 1. Symptomatic regrowth 2. Pain at 3 & 6 weeks (VAS) 3. Return to shoe wear 4. Return to normal activity 5. Number of dressings |
|  |  |  |  | B: Chemical ablation with Sodium Hydroxide (n=45) |  |
| Tatlican (2009) [20] | RCT (Turkey) | 24 months | 110 participants (148 procedures); Mean age 31.6-32.7 years. 54/56 M/F;  Grade 2 (n=65) or 3 (n=83) ingrowing nail. | A: Partial nail avulsion with 1 minute phenol cauterisation (n=37) | 1. Recurrence at 6-month intervals for 24 months 2. Healing time. Examined on alternate days until healing achieved 3. Pain at 2^nd^, 10^th^, 16^th^, 24^th^ & 30^th^ days of follow up 4. Drainage and tissue damage at 2^nd^, 10^th^, 16^th^, 24^th^ & 30^th^ days of follow up |
|  |  |  |  | B: Partial nail avulsion with 2-minute phenol cauterisation (n=36) |  |
|  |  |  |  | C: Partial nail avulsion with 3-minute phenol cauterisation (n=37) |  |
| Uygur (2016) [47] | Prospective RCT (Turkey) | 6 months | 128 participants; Mean age 22.8 (range 12-48 years); 83/41 M/F; Heifetz stage I (n= 28, 23%), Stage I (n=73, 58%), Stage III (n=23, 18%) | A: Winograd procedure and new suturing technique (n=64) | 1. Recurrence at 2 weeks, 1 and 6 months 2. Satisfaction at suture removal, 1 and 6 months 3. Time elapsed before shoes could be worn at 2 weeks, 1 and 6 months 4. Return to work/school |
|  |  |  |  | B: Winograd procedure and traditional suturing technique (n=64) |  |
| Van der Ham (1990) [39] | RCT (Netherlands) | 14 months | 249 participants; Age range 3-97 years; 158/ 91 M/F; Severity not reported | A: Wedge excision (n=124) | 1. Analgesic required after 7 days and weekly until healed for 14 months 2. Sick leave 3. Recurrence for 14 months 4. Re-operation 5. Nail Spikes 6. Healing time 7. Time required off work |
|  |  |  |  | B: Segmental phenol cauterisation (n=125) |  |
| Varma (1983) [26] | RCT (UK) | 6 months | 67 participants; Age, gender and severity not reported | A: Surgical wedge excision (n=35) | 1. Symptomatic recurrence at 3 and 6 months 2. Healing time at 1 week, 1 and 3 months |
|  |  |  |  | B: Phenol wedge cauterisation (n=28) |  |
| Wallace (1979) [24] | Prospective RCT (UK) | 15 months | 68 participants; Age range 10 to 73 years; gender and severity not reported | A: Gutter treatment (n=32) | 1. Number of successes/Number of failures 2. Number having no further operation/ Number undergoing reoperation 3. Pain after 1 day |
|  |  |  |  | B: Wedge resection (n= 36) |  |
| M/F, Male/Female; VAS, Visual Analogue Scale; RCT, Randomised Controlled Trial; QOL, Quality of Life, NaOH, Sodium Hydroxide | | | | | |
